# Differential expression of genes in salivary glands of male *Rhipicephalus *(*Boophilus*)*microplus *in response to infection with *Anaplasma marginale*

**DOI:** 10.1186/1471-2164-11-186

**Published:** 2010-03-18

**Authors:** Zorica Zivkovic, Eliane Esteves, Consuelo Almazán, Sirlei Daffre, Ard M Nijhof, Katherine M Kocan, Frans Jongejan, José de la Fuente

**Affiliations:** 1 Department of Infectious Diseases and Immunology, Utrecht Centre for Tick-borne Diseases (UCTD), Faculty of Veterinary Medicine, Utrecht University, Yalelaan 1, 3584CL, Utrecht, the Netherlands; 2 Departamento de Parasitologia, Instituto de Ciências Biomédicas, Universidade de São Paulo, Av. Prof. Lineu Prestes, 1374, CEP 05508-900, São Paulo, Brazil; 3 Facultad de Medicina Veterinaria y Zootecnia, Universidad Autónoma de Tamaulipas, Km.5 carretera Victoria-Mante, CP 87000 Cd. Victoria, Tamaulipas, Mexico; 4 Department of Veterinary Pathobiology, Center for Veterinary Health Sciences, Oklahoma State University, Stillwater, OK 74078, USA; 5 Department of Veterinary Tropical Diseases, Faculty of Veterinary Science, University of Pretoria, Private Bag X04, 0110, Onderstepoort, South Africa; 6 Instituto de Investigación en Recursos Cinegéticos IREC (CSIC-UCLM-JCCM), Ronda de Toledo s/n, 13005 Ciudad Real, Spain

## Abstract

**Background:**

Bovine anaplasmosis, caused by the rickettsial tick-borne pathogen *Anaplasma marginale *(Rickettsiales: Anaplasmataceae), is vectored by *Rhipicephalus *(*Boophilus*)*microplus *in many tropical and subtropical regions of the world. *A. marginale *undergoes a complex developmental cycle in ticks which results in infection of salivary glands from where the pathogen is transmitted to cattle. In previous studies, we reported modification of gene expression in *Dermacentor variabilis *and cultured *Ixodes scapularis *tick cells in response to infection with *A. marginale*. In these studies, we extended these findings by use of a functional genomics approach to identify genes differentially expressed in *R. microplus *male salivary glands in response to *A. marginale *infection. Additionally, a *R. microplus*-derived cell line, BME26, was used for the first time to also study tick cell gene expression in response to *A. marginale *infection.

**Results:**

Suppression subtractive hybridization libraries were constructed from infected and uninfected ticks and used to identify genes differentially expressed in male *R. microplus *salivary glands infected with *A. marginale*. A total of 279 ESTs were identified as candidate differentially expressed genes. Of these, five genes encoding for putative histamine-binding protein (22Hbp), von Willebrand factor (94Will), flagelliform silk protein (100Silk), Kunitz-like protease inhibitor precursor (108Kunz) and proline-rich protein BstNI subfamily 3 precursor (7BstNI3) were confirmed by real-time RT-PCR to be down-regulated in tick salivary glands infected with *A. marginale*. The impact of selected tick genes on *A. marginale *infections in tick salivary glands and BME26 cells was characterized by RNA interference. Silencing of the gene encoding for putative flagelliform silk protein (100Silk) resulted in reduced *A. marginale *infection in both tick salivary glands and cultured BME26 cells, while silencing of the gene encoding for subolesin (4D8) significantly reduced infection only in cultured BME26 cells. The knockdown of the gene encoding for putative metallothionein (93 Meth), significantly up-regulated in infected cultured BME26 cells, resulted in higher *A. marginale *infection levels in tick cells.

**Conclusions:**

Characterization of differential gene expression in salivary glands of *R. microplus *in response to *A. marginale *infection expands our understanding of the molecular mechanisms at the tick-pathogen interface. Functional studies suggested that differentially expressed genes encoding for subolesin, putative von Willebrand factor and flagelliform silk protein could play a role in *A. marginale *infection and multiplication in ticks. These tick genes found to be functionally relevant for tick-pathogen interactions will likely be candidates for development of vaccines designed for control of both ticks and tick-borne pathogens.

## Background

Bovine anaplasmosis, caused by the obligate intracellular rickettsial pathogen, *Anaplasma marginale *(Rickettsiales: Anaplasmataceae), is characterized in cattle by anemia, fever, weight loss and reduced milk production [1]. Transmission of *A. marginal*e occurs mechanically by biting flies and blood-contaminated fomites, while ticks are biological vectors [2]. Approximately 20 tick species have been incriminated worldwide as vectors of *A. marginale *[2]. Of these tick species, the southern cattle tick, *Rhipicephalus *(*Boophilus*)*microplus*, found in tropical and subtropical regions of the world, is considered to be the most economically important ectoparasite of livestock [3]. *R. microplus *vectors several pathogens and *A. marginale *is among the most important, causing notable economic loss in milk and beef production [4].

The developmental cycle of *A. marginale *was described in *Dermacentor *ticks, and male ticks were shown to become persistently infected with *A. marginale *and able to transmit infection repeatedly when transferred among cattle [5]. The *A. marginal*e development, multiplication in the tick and transmission to the vertebrate host are coordinated with tick feeding. Within *Dermacentor *ticks, *A. marginale *undergoes a complex developmental cycle that begins with the infection of the gut cells. After a second tick feeding, many other tick tissues become infected, including the salivary glands from where the *A. marginale *is transmitted to cattle [6]. Although the developmental cycle of *A. marginale *has not been described for *Rhipicephalus *(*Boophilus*) spp., the developmental cycle in *R. microplus *is most likely similar and males may also play an important role in pathogen transmission [7].

Molecular interactions at the tick-pathogen interface ensure survival and development of both the pathogen and vector. While recent studies on several pathogens have demonstrated that tick gene expression is modified in response to pathogen infection [8-10], information on the function of the differentially expressed genes is limited [11]. RNA interference (RNAi) has been shown to be a useful tool for the characterization of the function of genes involved in tick-host-pathogen interactions and the transmission of tick-borne pathogens and for screening for tick protective antigens [11]. Recently, genes differentially expressed in cultured IDE8 tick cells in response to *A. marginale *infection were identified and their impact on pathogen infection in *D. variabilis *ticks was characterized by RNAi during the pathogen developmental cycle [11].

Tick cell lines have been used successfully to study vector-pathogen interactions [12]. However, these studies were conducted in the IDE8 and ISE6 tick cell lines derived from *Ixodes scapularis *embryos which is not a natural vector of *A. marginale *[12]. Recently, a Brazilian isolate of *A. marginale *was propagated successfully in the BME26 cell line derived from *R. microplus *[13] which provided the opportunity to study the *A. marginale*-tick interface in the cells cultured from a natural tick vector.

The objective of this study was to identify *R. microplus *genes differentially expressed in male salivary glands in response to infection with *A. marginale *by using suppression subtractive hybridization (SSH) approach and to characterize the function of those genes by RNAi. SSH enables identification of low-abundant rare transcripts through the comparison of two cDNA populations by selective amplification of the genes expressed in one population but not in the other [14,15]. The results of these SSH studies were validated by real-time RT-PCR in *R.microplus *ticks and cultured BME26 tick cells for selected genes. Finally, functional analyses were conducted on selected genes by RNAi in both *R. microplus *male ticks and cultured BME26 cells to determine the putative role of these genes in *A. marginale*-tick interactions.

## Results

### Identification of differentially expressed genes in *R. microplus *male salivary glands

**Figure 1:**
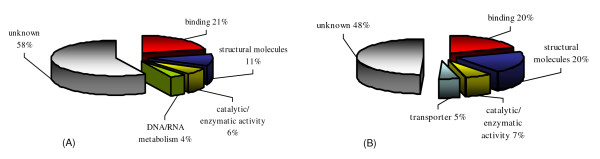
**Gene ontology assignments of ESTs differentially expressed in *R. microplus *male salivary glands in response to *A. marginale *infection**. (A) Genes up-regulated in infected salivary glands. (B) Genes down-regulated in infected salivary glands.

Two SSH libraries, forward and reverse, were constructed to identify genes up- and down-regulated in *R. microplus *male salivary glands in response to *A. marginale *infection. From each library, 288 randomly selected clones were identified and sequenced. After removing vector sequences and eliminating EST clones with poor sequence quality, datasets of 128 and 151 ESTs from forward and reverse subtracted libraries were obtained, respectively, and used for bioinformatics analyses. Clustering and assembly of ESTs from forward subtracted library (up-regulated in infected cells) resulted in 43 unique transcripts of which 10 were derived from two or more ESTs (consensus sequences) and 33 were derived from a single EST (singletons). Assembly of the ESTs in the reverse subtracted library (down-regulated in infected cells) yielded 56 unique sequences (24 consensus sequences and 32 singletons). Automated annotation was then used to search public domain protein databases for putative functions (Additional file 1: Table S1). Gene ontology assignments demonstrated that up-regulated genes encoded for proteins with putative functions of binding (21%), structural molecules (11%), catalytic/enzymatic activity (6%), DNA/RNA metabolism (4%), and 58% had no known function (Figure 1A). Putative functions assigned to down-regulated genes included binding (20%), structural molecules (20%), catalytic/enzymatic activity (7%), transport (5%) and 48% had no known function (Figure 1B).

### Differential gene expression in *A. marginale*-infected *R. microplus *male salivary glands and cultured BME26 cells

**Figure 2:**
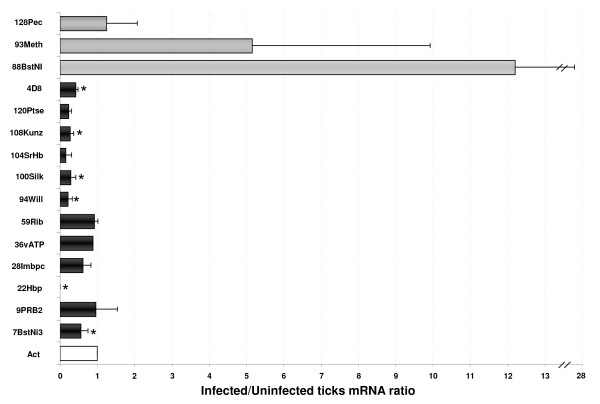
**Differential gene expression in *A. marginale*-infected *R. microplus *male salivary glands**. Real-time RT-PCR was done on uninfected and infected pooled salivary glands (two independent experiments). Genes up-regulated (white bars) and down-regulated (black bars) in infected salivary glands are shown. Bars represent average + SD mRNA ratios. The mRNA levels were normalized against tick β-actin using the comparative Ct method. The mRNA levels were compared between infected and uninfected tick salivary glands by Students's *t *test (*p ≤ 0.05). Gene IDs are described in additional file 2: Table S2.

Fourteen candidate genes with putative functions in tick-pathogen interactions were selected for validation of SSH results by real-time RT-PCR (Additional file 2: Table S2). Real-time RT-PCR analyses were done on the same pooled RNA samples used for SSH. Statistically significant differences in expression were obtained for 5 genes (Figure 2). Similar to the SSH results, genes encoding for putative female-specific histamine-binding protein (22Hbp), flagelliform silk protein (100Silk), Kunitz-like protease inhibitor precursor (108Kunz), and proline-rich protein BstNI subfamily 3 precursor (7BstNI3) were significantly down-regulated in infected tick salivary glands. The gene encoding for the putative von Willebrand factor (94Will), identified to be up-regulated by SSH, was shown by real-time RT-PCR to be significantly down-regulated in the infected tick salivary glands. For the other 9 genes, mRNA levels were not significantly different between infected and uninfected ticks. Subolesin (4D8), used as a positive control, was down-regulated in *A. marginale*-infected tick salivary glands.

**Figure 3:**
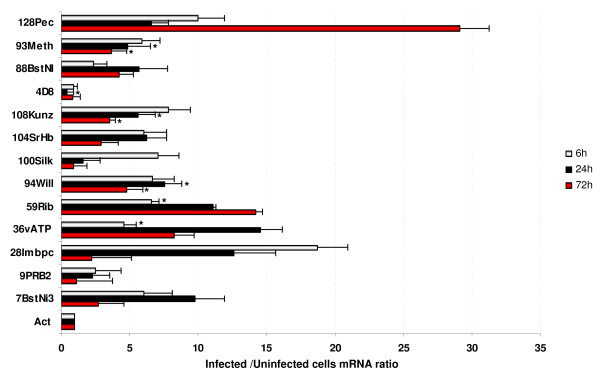
**Differential gene expression in *A. marginale*-infected cultured BME26 tick cells**. Real-time RT-PCR was done on uninfected and infected BME26 cells collected at 6, 24 and 72 hpi (four independent cultures each). Bars represent average + SD mRNA ratios. The mRNA levels were normalized against tick β-actin using the comparative Ct method. The mRNA levels were compared between infected and uninfected tick cells by Students's *t *test (*p ≤ 0.05). Gene IDs are described in additional file 2: Table S2.

To evaluate the use of cultured BME26 cells for studying *A. marginale*-tick interactions, the same primers were used for real-time RT-PCR analysis of uninfected and *A. marginale*-infected BME26 tick cells. Twelve of the 14 selected genes were amplified from BME26 cultured cells. Gene expression profiles were studied for each gene at 6, 24 and 72 hours post-infection (hpi) and compared with uninfected cells collected at the same time points (Figure 3). Genes encoding for putative vacuolar H+-ATPase (36vATP) and ribosomal protein S29 (59Rib) were significantly up-regulated at 6 hpi, while putative Kunitz-like protease inhibitor precursor (108Kunz), metallothionein (93 Meth) and von Willebrand factor (94Will) were significantly up-regulated 24 and 72 hpi. The mRNA levels for the rest of the genes were not statistically different between infected and uninfected BME26 cells. The subolesin (4D8) control was significantly downregulated by 24 hpi.

All the tick sequence-derived primers were tested against bovine RNA by RT-PCR. Amplicons were not obtained for any of the primer pair tested (data not shown).

### Functional roles of genes differentially expressed *in R. microplus *ticks and cultured BME26 cells in response to infection with *A. marginale*

The five genes corroborated by real-time RT-PCR to be differentially expressed in *A. marginale *infected tick salivary glands, female-specific histamine-binding protein (22Hbp), flagelliform silk protein (100Silk), Kunitz-like protease inhibitor precursor (108Kunz), proline-rich protein BstNI subfamily 3 precursor (7BstNI3) and von Willebrand factor (94Will) (Figure 2), were selected for functional analyses in ticks. The effect of gene knockdown on *A. marginale *infection and multiplication in *R. microplus *male tick salivary glands was evaluated by RNAi. The mRNA levels after RNAi were reduced for putative von Willebrand factor (94Will), flagelliform silk protein (100Silk) and subolesin (4D8), while silencing of the other genes did not result in statistically significant differences from the control (Table 1).

**Table 1:** *A. marginale *infection levels in tick salivary glands after RNAi and tick mortality rates in dsRNA-injected *R. microplus *males.

Experimental group	Gene expression silencing (%) ± SD^a^	*A. marginale *infection levels (% change with respect to controls)^b^	Mortality rate^c^
94Will	85.6 ± 16.9	29.4 ± 0.6 (-63%)	80%**

100Silk	89.8 ± 9.4	17.0 ± 1.1 (-79%)*	74.3%**

Subolesin (4D8)	82.7 ± 12.7	32.0 ± 1.6 (-60%)	68.6%**

Control	--	80.0 ± 1.9	57.1%

^a^Total RNA was extracted from infected tick salivary glands after RNAi and analyzed by real-time RT-PCR to determine gene expression silencing with respect to control ticks injected with the GIII dsRNA.

^b^The *A. marginale *infection levels were analyzed by *msp4 *PCR, expressed as *msp4 *copies per tick ± SD and compared between test and control ticks injected with the GIII dsRNA by Student's *t *test (*p < 0.05).

^c^Tick mortality was evaluated as the ratio of dead male ticks 7 days after dsRNA injection to the total number of the attached male ticks, and was compared between test and control ticks injected with the GIII dsRNA by *χ*^2^-test (**α < 0.025).

The effect of RNAi of selected genes on male *B. microplus *mortality was determined. Tick mortality was significantly higher in groups injected with dsRNA for von Willebrand factor (94Will), flagelliform silk protein (100Silk) and subolesin when compared with the unrelated dsRNA-injected control ticks (Table 1). Despite the fact that individual variation in gene expression affected the statistical significance of results, silencing of the genes encoding for putative von Willebrand factor (94Will), flagelliform silk protein (100Silk) and subolesin (4D8) resulted in 63%, 79% and 60% decrease in *A. marginale *infection levels in *R. microplus *male salivary glands, respectively (Table 1). Characterization of the effect of gene knockdown on *A. marginale *infections in cultured BME26 tick cells was attempted for all the genes which were shown to be differentially expressed in tick cells and/or tick salivary glands. However, mRNA levels were reduced only for putative ribosomal protein S29 (59Rib), metallothionein (93 Meth), flagelliform silk protein (100Silk) and subolesin (4D8) genes (Table 2). Of these genes, two genes encoding for putative flagelliform silk protein (100Silk) and subolesin resulted in 12% and 17% reduction of *A. marginale *infection levels, respectively, when compared with controls (Table 2). The knockdown of the gene encoding for putative metallothionein (93 Meth) resulted in higher *A. marginale *infection levels in tick cells (Table 2).

**Table 2:** *A. marginale *infection levels in cultured BME26 tick cells after RNAi.

Experimental group	Silencing of gene expression (%) ± SD^a^	*A. marginale *infection levels (% change with respect to controls)^b^
59Rib	71.7 ± 31.1	8.6 ± 0.5 (-3%)

93 Meth	65.0 ± 14.4	18.0 ± 0.0 (+102%)*

100Silk	99.5 ± 0.3	7.8 ± 0.6 (-12%)*

Subolesin (4D8)	88.1 ± 7.1	7.4 ± 0.3 (-17%)*

Control	**--**	8.9 ± 1.4

^a^Total RNA was extracted from infected tick cells after RNAi and analyzed by real-time RT-PCR to determine gene expression silencing with respect to control cells treated with buffer only.

^b^The *A. marginale *infection levels were analyzed by *msp4 *PCR, expressed as *msp4 *DNA (ng) ± SD and compared between test and control ticks by Student's *t *test (*p < 0.05).

## Discussion

In the present study we identified *R. microplus *male salivary gland genes differentially expressed in response to *A. marginale *infection by use of SSH and real-time RT-PCR. Development and multiplication of *A. marginale *in salivary gland cells involves molecular interactions between pathogen- and tick-derived molecules. Salivary gland, the tissue of interest in this study, is a critical site in the developmental cycle from where the pathogen is transmitted to cattle. Recently, tick salivary gland proteins were shown to play a role in the infection and transmission of *Borrelia burgdorferi *[16,17], *A. phagocytophilum *[18] and *A. marginale *[19]. *A. marginale *membrane surface proteins involved in tick salivary gland colonization have been identified and partially characterized [20,21]. Understanding the molecular mechanisms of *A. marginale*-tick interactions for *R. microplus*, one of the most important vectors of *A. marginale *worldwide, is fundamental toward development of novel control measures [22].

Some of the genes identified by SSH, including those genes encoding for putative tick cement proteins, female specific histamine binding protein, IgG binding protein C, salivary gland-associated protein 64P, flagelliform silk protein and von Willebrand factor, were identified previously in different tick species and appear to be involved in tick feeding or pathogen infection [10,23-25]. However, most of the differentially expressed genes identified in this study have not been shown to be associated with tick-pathogen interaction previously. Some cellular functions affected by *A. marginale *infection of *R. microplus*, such as cell structure and enzymatic processes, were reported previously in infected tick IDE8 cultured cells [11]. The discrepancy observed for some studied genes between SSH and real-time RT-PCR results may reflect differences between both methods for identifying differentially expressed genes or the presence of multiple sequences targeted during RT-PCR reactions that affect the results of mRNA quantification for some genes.

In a recent study, genes differentially expressed in cultured IDE8 tick cells in response to *A. marginale *infection were identified and functional studies conducted in *D. variabilis *suggested that these genes may play different roles during pathogen infection, development and trafficking from midguts to salivary glands [11]. Some of the genes identified by de la Fuente et al. [11] such as gluthathione S-tranferase, selenoprotein M and ferritin were also shown to be differentially expressed in *R. microplus *salivary glands in response to *A. marginale *infection. However, these genes were absent from the current EST dataset which could be due to differences in the system used for EST discovery (cultured IDE8 tick cells versus *R. microplus *salivary glands) and/or other factors such as tick species and/or *A. marginale *strain and infection levels.

While tick cell lines have been used successfully in *A. marginale *functional genomics studies [11], this is the first report of the use of the BME26 tick cell line derived originally from a natural vector of *A. marginale *for functional studies of tick-pathogen interactions. Since these studies were conducted on ticks and tick cells of the same species, most of the genes identified in tick salivary glands were also amplified from cultured BME26 tick cells. However, expression profiles of selected genes observed in cultured BME26 cells were not identical to that found in tick salivary glands. For example, the expression of the putative von Willebrand factor (94Will) was down-regulated in tick salivary glands but up-regulated in cultured BME26 tick cells infected with *A. marginale*. These differences may have resulted from tissue-specific regulation of gene expression or because we only observed early stages of infection in the cultured BME26 tick cells (6-72 hpi). As reported previously [11], results of studies using cultured tick cells must be validated in naturally infected ticks. Interestingly, expression of putative vacuolar H+-ATPase (36vATP) was significantly up-regulated in *A. marginale*-infected cultured BME26 cells, as reported for previous gene expression studies of cultured IDE8 cells in response to *A. marginale *infection [11].

RNAi was used in this study to assign the effect of selected gene knockdown on *A. marginale *infection and multiplication in ticks. Although statistically significant for flagelliform silk protein (100Silk) only, results of RNAi experiments suggested that putative von Willebrand factor (94Will), flagelliform silk protein (100Silk) and subolesin could play a role in pathogen infection of *R. microplus *male salivary glands. RNAi experiments in cultured BME26 tick cells provided further evidence that flagelliform silk protein (100Silk) and subolesin may play a role in *A. marginale *infection and/or multiplication in tick cells and suggested that metallothionein (93 Meth) may be involved tick defense against pathogen infection.

The flagelliform silk protein was identified previously in tick and orb weaving spider salivary glands but its function was not linked to pathogen infection [26-28]. Mulenga et al. [29] demonstrated that the flagelliform silk protein may be involved in tick attachment. In previous studies of *I. ricinus *after *B. burgdorferi *infection, the von Willebrand factor was isolated from tick salivary glands and shown to be up-regulated but its possible role in infection was not studied [10]. A von Willebrand factor-like motif is present in the major hemelipoglycoprotein found in ixodid ticks and this protein has been shown to play a role as a heme-sequestering factor during tick feeding [30]. Therefore, silencing of these genes may affect tick feeding, mortality and development of *A. marginale *in salivary glands. However, as shown previously for subolesin [19], gene expression studies in cultured BME26 tick cells have provided evidence that that the flagelliform silk protein may play a role in the infection of ticks with *A. marginale*.

The results for gene expression and silencing of subolesin in *R. microplus *male salivary glands and cultured BME26 cells infected with *A. marginale *reported herein are in agreement with previous studies in which subolesin knockdown reduced *A. marginale *infection in *D. variabilis *and cultured IDE8 cells [11,19]. Subolesin, discovered as a tick protective antigen in *I. scapularis*, has been shown to be conserved in many tick species [31,32]. Subolesin was shown by both RNAi gene knockdown and immunization trials using the recombinant protein to protect vertebrate hosts against tick infestations, reduce tick survival and reproduction, and cause degeneration of gut, salivary gland, reproductive tissues and embryos [31-37]. Targeting of subolesin by RNAi or vaccination also decreased tick vector capacity for *A. marginale *and *A. phagocytophilum *[19]. In addition, subolesin was shown to function in the control of gene expression in ticks [38,39] and to be differentially expressed in *Anaplasma*-infected ticks and cultures tick cells [11,40]. However, subolesin expression in *R. microplus *tick salivary glands and cultured BME26 cells was different to previous reports showing up-regulation in *A. marginale*-infected *D. variabilis *salivary glands and cultured IDE8 cells [41]. These differences could be due to tick species-specific differences in gene regulation or to other factors such as pathogen strain and infection levels. Nonetheless, these results expanded our knowledge on the role of subolesin in tick-*Anaplasma *interactions.

Metallothioneins are a family of low molecular weight proteins with a high affinity for divalent metals that function in cell detoxification, apoptosis, stress response and immunity [41-43]. Metallothioneins control the cellular zinc ion levels, which are known to be important in the immune system, and their expression has been associated with protective response against pathogens [44-48]. The results suggested a role for tick metallothioneins in defense against bacterial infections. Interestingly, selenoproteins that regulate the levels of another important trace mineral in the organism were suggested to participate in the cellular response to limit *A. marginale *infection in tick cells [11].

Although dsRNA sequences used in this study do not contain any significant overlap with other known *R. microplus *genes, the possibility of off-target gene silencing effects cannot be excluded due to the limited amount of sequence data available. However, RNAi seems to be very sequence-specific in ticks with little off-target effects [38]. Availability of the complete *R. microplus *genome sequence data will facilitate screening for potential off-target effects. These can subsequently be minimized by avoiding the use of dsRNAs or siRNAs containing sequences which are present in multiple genes.

In our study *R. microplus *male salivary gland genes differentially expressed in response to *A. marginale *infection were identified by using SSH approach. Recently a *R. microplus *microarray (NimbleGen) has been developed and used for the analysis of acaricide- inducible genes in *R. microplus *[49]. Microarray chip hybridization could be an alternative approach for identifying *R. microplus *differentially expressed genes in response to *A. marginale *infection.

## Conclusions

In this study, *A. marginale *infection of *R. microplus *was shown to modulate in male salivary glands the expression of genes encoding for putative proteins involved in binding, catalytic/enzymatic activity, transport, DNA/RNA metabolism and structural molecules. Five genes encoding for putative histamine-binding protein (22Hbp), von Willebrand factor (94Will), flagelliform silk protein (100Silk), Kunitz-like protease inhibitor precursor (108Kunz) and proline-rich protein BstNI subfamily 3 precursor (7BstNI3) were confirmed by real-time RT-PCR to be down-regulated in tick salivary glands infected with *A. marginale*. Functional studies suggested that differentially expressed genes encoding for subolesin, putative von Willebrand factor and flagelliform silk protein could play a role in *A. marginale *infection and multiplication in ticks. Additionally, for the first time RNAi in cultured BME26 tick cells was used to study *A. marginale*-tick molecular interactions and suggested that subolesin and flagelliform silk protein may be required by *A. marginale *for infection and multiplication in these cultured cells. Collectively these data are important toward understanding the molecular mechanisms involved in *R. microplus*-*A. marginale *interactions.

## Methods

### Experiment design

A suppression subtractive hybridization (SSH) approach was used to identify genes differentially expressed in *R. microplus *male salivary gland genes in response to *A. marginale *infection. Sequences obtained by SSH were used to search for homology/identity to nucleotide and protein databases. Real-time RT-PCR was used to validate differential expression of selected genes in uninfected and *A. marginale-*infected *R. microplus *salivary glands. Differential expression patterns of selected genes were also studied in cultured BME26 cells at 6, 24 and 72 hpi by real-time RT-PCR. The genes that proved to have significantly different mRNA levels between uninfected and *A. marginale*-infected ticks or cultured BME26 tick cells were then selected for functional studies. RNAi was used to characterize the function of selected genes in *A. marginale *infection *in vivo *in *R. microplus *male ticks and *in vitro *in cultured BME26 tick cells.

### *Rhipicephalus microplus *ticks

The *R. microplus *ticks used for construction of the SSH libraries originated from Mozambique and were provided by ClinVet International (Pty), Bloemfontein, South Africa. The *R. microplus *(Susceptible, CENAPA, Mexico strain) ticks used for the RNAi experiments were obtained from laboratory colonies maintained at the University of Tamaulipas, Mexico. Originally, these tick strains were collected from infested cattle in Tapalpa, Jalisco, Mexico. The ticks were maintained on cattle at the tick rearing facilities at the Utrecht Center for Tick-Borne Diseases, Utrecht University or the University of Tamaulipas. Larvae were kept off-host in an incubator at 20°C with 95% relative humidity and 12 hours light: 12 hours dark photoperiod. Cattle were cared for in both The Netherlands and Mexico in accordance with standards specified in the Guide for Care and Use of Laboratory Animals.

### Tick cell line

The tick cell line BME26 was derived originally from embryos of *R. microplus *following the protocol established by Kurrti et al[50]. The cells were maintained in L-15B300 medium [51] supplemented with 5% heat-inactivated FBS (Gibco/Invitrogen, NY, USA), 10% TPB (Difco, Detroid, MI, USA), 0,1% bovine lipoprotein (ICN, Irvine, CA), 100 units ml^-1 ^penicillin (Gibco/Invitrogen) and 100 μg ml^-1 ^streptomycin (Gibco/Invitrogen) at pH 7.2. Cultures were grown at 34°C in 25 cm^2 ^plastic flasks (Nalge Nunc Int. Rochester, NY) containing 5 ml of the complete medium, which was changed weekly. Monolayers were subcultured when they reached a density of approximately 10^7 ^cells/ml and approximately 8 × 10^5 ^cells/ml were transferred to the new flask.

### *A. marginale *isolates

The *A. marginale *isolate used for infection of *R. microplus *ticks and construction of SSH libraries was obtained from an infected calf in Texas, USA, in 1977. The isolate was subsequently passaged in splenectomized calves and blood samples were collected at the peak parasitemia (40%), prepared as a stabilates with 10% DMSO and stored in 2 ml aliquots in liquid nitrogen. A Brazilian strain of *A. marginale *with an inclusion appendage (UFMG1) [52], which was recently established and propagated in IDE8 tick cells, was used to infect BME26 cells [14].

### Uninfected and *A. marginale*-infected *R. microplus *male ticks for SSH libraries construction

To obtain *A. marginale*-infected *R. microplus *male ticks, eight month-old non-splenectomized, tick-naive Holstein-Friesian calf (No.4280) was infected intravenously with the Texas isolate of *A. marginale*. *R. microplus *larvae were then fed on the calf with ascending parasitemia. Approximately 200 partially fed male ticks were collected after 21 days of feeding and the presence of *A. marginale *infection in salivary glands was confirmed in 20 individually dissected tick salivary glands by *msp4 *PCR [53]. Salivary glands from the remaining ticks were dissected in cold PBS, pooled and immediately stored in TriReagent (Sigma-Aldrich, Zwijndrecht, The Netherlands) at -80°C. Uninfected ticks were fed in a similar way on an uninfected calf and the male tick salivary glands were dissected and stored as described previously. Cattle and tick feeding studies were conducted in accordance with approval of the Animal Experiments Committee (DEC) of the Faculty of Veterinary Medicine, Utrecht University (DEC No. 0604.0801).

### Uninfected and *A. marginale*-infected cultured BME26 tick cells

The tick cell line BME26 was cultured as described above. Approximately 4 × 10^6 ^cells from the passage 72 were plated in 24-well plates (Nunc) and maintained in *Anaplasma *medium [54] at 34°C for 3 days. The cells were infected with the 15 day-old culture of *A. marginale *in BME26 cells. Five milliliters of this suspension were transferred to a plastic tube. The tube was immersed in liquid nitrogen for 5 min for cell disruption and rickettsia releasing, followed by thawing in a water bath at 37°C. Approximately 500 μl of infected cell suspension was inoculated into each well onto uninfected BME26 monolayer. The plate was maintained at 34°C and cells harvested at 6, 24 and 72 hpi from 4 wells for each time point. Uninfected control cells (n = 4 wells) were cultured in the same way but with the addition of 500 μl of *Anaplasma *medium only and the cells were harvested at the same time points. Cells were centrifuged at 800 g for 10 min and RNA/DNA was isolated using Trizol reagent (Gibco/Invitrogen), as recommended by the manufacturer. The infection of the tick cells by *A. marginale *was corroborated by *msp4 *PCR [53].

### Suppression-subtractive hybridization

Total RNA was isolated from uninfected and *A. marginale*-infected tick salivary glands using Tri reagent (Sigma-Aldrich) following the manufacturer's protocol. RNA quality was checked by gel electrophoreses to confirm integrity of RNA preparations. Pools of 2 μg of total RNA were made from uninfected and *A. marginale*-infected salivary glands. The cDNA synthesis was done using the Super SMART PCR cDNA synthesis kit (Clontech-Takara, Saint-Germain-en-Laye, France), a method for producing high quality cDNA from a low amount of starting material. The cDNA was then directly used for PCR select subtraction (Clontech-Takara) based on a technique previously described by Diatchenko et al. [14,15] and SSH libraries were constructed according to manufacturer's instructions. The double stranded cDNA from both groups (infected and uninfected salivary glands) was *Rsa*I digested. Part of the digested cDNA was ligated with Adapter 1 and part with the Adapter 2R, and the rest was saved for use as a driver in preparation for hybridization. The forward subtracted library was made by hybridizing adapter ligated cDNA from *A. marginale-*infected tick salivary glands as the tester in the presence of uninfected tick salivary gland CDNA as the driver. This forward reaction library was designed to produce clones that are overexpressed or up-regulated in infected salivary glands. The reverse library was made in the same way but in this case the adapter ligated cDNA from uninfected tick salivary glands was used as the tester and infected salivary gland cDNA as the driver. The reverse reaction library was designed to produce clones underexpressed or down-regulated in infected salivary glands. In either case the driver cDNA was added in excess during each hybridization to remove common cDNAs by hybrid selection and leaving over expressed and novel tester cDNA to be recovered and cloned. Differentially expressed cDNAs were PCR amplified with Advantage PCR polymerase mix (Clontech-Takara), cloned using pGEM Easy T/A cloning kit (Promega, Madison, WI, USA), transformed into JM109 and plated on LB with ampicillin, X-gal and IPTG. Approximately 300 clones were randomly picked up from each plated library in 96- well plates containing LB medium supplemented with ampicillin and grown overnight. Plasmids were purified using Wizard SV 96 Plasmid DNA purification system (Promega). Plasmid inserts were PCR amplified and PCR products were fully sequenced in an accredited service laboratory (BaseClear, Leiden, The Netherlands) using vector specific primers.

### Sequence analysis and database search

Partial sequences were obtained for 279 out of 576 randomly selected sequenced SSH library clones (288 from each forward- and reverse-subtracted libraries). The cDNA Annotation System software (CAS; Bioinformatics and Scientific IT Program (BSIP), Office of Technology Information Systems (OTIS), National Institute of Allergy and Infectious Diseases (NIAID), Bethesda, MD, USA) http://exon.niaid.nih.gov was used for automated sequence clean up, contig assembly, Blast analysis [55] against multiple sequence databases (nonredundant sequence database and databases of tick-specific sequences. (http://www.vectorbase.org/index.php and BmGI2; http://compbio.dfci.harvard.edu/), identification and locating of signal peptide cleavage sites and gene ontology (GO) assignments. Genes for further analyses were annotated by manual curation.

### Nucleotide sequence accession numbers

The gene sequences reported in this paper are deposited in the GenBank Data Library under accession numbers: GO496166-G0496262.

### Real-time RT- PCR analysis

The same RNA samples prepared before for SSH from uninfected and *A. marginale-*infected tick salivary glands were used for real-time RT-PCR. Total RNA was extracted as described above from uninfected and *A. marginale*-infected cultured BME26 tick cells from quadruplicate cultures at 6, 24 and 72 hpi and used for real-time RT-PCR. Oligonucleotide primers were synthesized based on the sequences determined for candidate differentially expressed genes (Table 3) and used in 25 μl RT-PCR reactions performed using the iScript one step RT-PCR kit with SYBR green (Bio-Rad, Hercules, CA, USA) and a Bio-Rad iQ5 thermal cycler following the manufacturer's protocol. The mRNA levels were normalized against tick β-actin (Genbank accession number AY255624) using the comparative Ct method [56]. The mRNA levels were compared between infected and uninfected tick salivary glands and tick cells by Student's *t *test (p = 0.05). Total RNA was isolated from bovine blood and RT-PCR reactions were performed using the oligonucleotide primers and conditions described in Table 3. PCR products were electrophoretically separated using 1.5% agarose gel stained with ethidium bromide for visualization.

**Table 3:** Real-time RT-PCR oligonucleotide primers and conditions.

EST	Upstream/downstream primer sequences (5'-3')	PCR annealing conditions
7BstNI3	AAACTGGGGAATCCAAAAGG GGGGTTTGGGATAGGGTTC	55°C/30 s

9PRB2	AACGACCGCCCAAAAATAAC AATTTGTTCCGGTTTTGTTCC	55°C/30 s

22Hbp	GGAGGTTACGAACTATGGGC ATGAGTTGGCAGTGCCTTAG	55°C/30 s

28ImbpC	CGGTACCATGATGCACTTTG TGATGGCGTCCCTAGTTACC	55°C/30 s

36vATP	GAAGGCTTCGAACAGAGTCG CTCAATTCTGGTGGCCAAG	55°C/30 s

59Rib	CCAGCAAGCGAGATTGTGTA GCGTACTGTCTGAAGCAACG	55°C/30 s

88BstNI	GTTTGGGGGCCTTAAGAAAA TTTTTCCCAAAAGGTTCTCC	55°C/30 s

93 Meth	CTGAACTGAACGCATCATGG GCACAACATTTTGCAGATGG	55°C/30 s

94Will	TCATTGACGAAGAAGCGATC TACAAGTCGCCCTGACACC	55°C/30 s

100Silk	TGAACCAGAGGGACCAACTC GTCTTGGACTCGGCAGTAGC	55°C/30 s

104SrHb	CGAACCCGAATGGATTATG TTCAAACATGAAGCGACAGC	55°C/30 s

108Kunz	ATGGAACTGTTCGGTTTTGC ATCCGCCGTAAATGAAGTTC	55°C/30 s

120Ptse	GCGCGACCTCTTTGTTAAAC CGAATACGCACAGAAGGTGAC	55°C/30 s

128Pec	AGGCCCAATTCTGATCTTTC CAAAGCTCAAACGTGTGGTG	55°C/30 s

Subolesin (4D8)	GAGACCAGCCCCTGTTCA CTGTTCTGCGAGTTTGGTAGATAG	54°C/30 s

Beta-actin	GACATCAAGGAGAAGCT(TC)TGC CGTTGCCGATGGTGAT(GC)	55°C/30 s

### RNA interference in ticks

Oligonucleotide primers containing T7 promoter sequences at the 5'-end were synthesized for *in vitro *transcription of dsRNA using the Acess RT-PCR system (Promega, Madison, WI, USA) and the Megascript RNAi kit (Ambion, Austin, TX, USA) following manufacturer's instructions. The dsRNA was purified and quantified by spectophotometry. Newly molted uninfected *R. microplus *males, were injected with approximately 0.3 μl of dsRNA (5 × 10^10 ^molecules/μl) in the lower right quadrant of the ventral surface of the exoskeleton of the tick [19]. Ticks (35/group) were injected using a Hamilton syringe with a 1 inch, 33 gauge needle. Control ticks were injected with *R. microplus *subolesin dsRNA (positive control) or the unrelated GIII dsRNA (negative control). The GIII sequence was identified in *R. microplus *and did not affect tick feeding, mortality and oviposition after RNAi (unpublished results). Ticks were held in a humidity chamber for 3-5 hours and mortality was recorded before the living ticks were allowed to feed in seven separate patches (five test genes and two controls), each one for a different group, glued on the back of a calf naturally infected with *A. marginale *in Tamaulipas, Mexico (approximately 4% rickettsemia during tick feeding). Ten females were placed in each patch simultaneously with injected males. Unattached ticks were removed 2 days after infestation and attached ticks were allowed to feed for 7 days. All the males were collected and salivary glands were dissected from individual ticks from each group. The salivary glands were stored in RNAlater (Qiagen) until used for DNA and RNA extraction to determine *A. marginale *infection levels by quantitative *msp4 *PCR [53] and to confirm gene knockdown by real-time RT-PCR. The mRNA levels of the target gene and the *A. marginale *infection were compared between test and control ticks by Student's *t*-test (p = 0.05). Tick mortality was evaluated as the ratio of the dead male ticks 7 days after dsRNA injection to the total number of attached male ticks feeding on the animal and was compared between test and control groups by χ^2^-test (α = 0.025).

### RNA interference in cultured BME26 tick cells

Approximately 5 × 10^5 ^BME26 cells/well were placed in 24-well plates (Nunc). The dsRNA prepared in the way described above for each of the 8 target genes was added to the culture wells (n = 4 for each treatment). Subolesin dsRNA was used as a positive control and control wells received elution buffer only. Each of the treated wells received 10 μl of dsRNA (5 × 10^10 ^molecules per microliter) and 190 μl of L15B *Anaplasma *medium and was incubated for 24 h. After this period, additional 300 μl of medium were added to each well. After 48 h cells were infected as described above. Three days post infection the cells were harvested from the plate, centrifuged at 800 × g for 10 min and used to extract RNA and DNA with TriReagent (Sigma). *A. marginale *infection levels were determined by *msp4 *PCR [53]. Gene expression silencing was confirmed by real-time RT-PCR using sequence-specific primers (Table 3) as described above.

## Authors' contributions

ZZ prepared SSH libraries, participated in sequence analyses, did the molecular work and drafted the manuscript. EE did the cell culture work and helped with drafting the manuscript. CA helped with the tick RNAi experiments and revision of the manuscript. AN helped with tick infection experiment and revision of the manuscript. SD and KK helped with drafting and revision of the manuscript. FJ and JF participated in design and coordination of the study and helped with drafting and revision of the manuscript. JF contributed to acquisition of molecular biology data. All authors read and approved the final manuscript.

## Supplementary Material

Additional file 1 Genes identified by SSH as differentially expressed in *A. marginale*-infected *R. microplus *male salivary glands. Click here for file

Additional file 2 Differentially expressed genes selected based on their putative role during *A. marginale *infection to validate SSH results by real-time RT-PCR. Click here for file
