# Supplementary material for: Differential expression of genes in salivary glands of male Rhipicephalus (Boophilus)microplus in response to infection with Anaplasma marginale
Source: BMC Genomics. 2010 Mar 18;11:186. doi: 10.1186/1471-2164-11-186 (PMC2848250; doi:10.1186/1471-2164-11-186)
Supplement: Additional file 1 — Genes identified by SSH as differentially expressed in A. marginale-infected R. microplus male salivary glands. [file 1471-2164-11-186-S1.DOC]

| **GenBank**  **Table S1** - Genes identified by SSH as differentially expressed in *A. marginale*-infected *R. microplus* male  salivary glands.  **Acc. No.** | **Best match to non-repetitive protein database and the *R. microplus* BmGI2 database** | **E-value** | **Molecular function** |
| --- | --- | --- | --- |
| **Up-regulated in infected salivary glands** | | | |
| GO496166 | metallothionein - *Oscillatoria brevis* (BAC76027.1)  b_microplus|TC23383 | 1^x^e^+00^ | binding |
| GO496167 | Von Willebrand factor - *Ixodes ricinus* (AAQ01562.1) | 2^x^e^-04^ | binding |
| GO496168 | salivary proline-rich protein - *Homo sapiens* (CAA30394.2) | 1^x^e^-14^ | binding |
| GO496169 | proline-rich protein BstNI subfamily 1 isoform 1 precursor - *Homo sapiens* (NP_005030.2) | 4^x^e^-25^ | binding |
| GO496170 | hydroxyproline-rich glycoprotein DZ-HRGP - *Volvox carteri f. nagariensis* (CAB62280.1) | 4^x^e^-23^ | binding |
| GO496171 | hypothetical protein isoform 2 - *Pan troglodytes* (XP_001151718.1) | 2^x^e^-25^ | binding |
| GO496172 | putative glycine-rich protein - *Arabidopsis thaliana* (CAA22153.1)  b_microplus|U92761 | 2^x^e^-46^ | binding |
| GO496173 | vacuolar ATP synthase subunit G-like protein - *Nasonia vitripennis* (XP_001600097.1)  b_microplus|TC24424 | 1^x^e^-19^ | binding |
| GO496174 | predicted protein - *Coprinopsis cinerea okayama* (EAU88308.1)  b_microplus|TC17118 | 6^x^e^-03^ | binding |
| GO496175 | shematrin-4 - *Pinctada fucata* (BAE93436.1)  b_microplus|CK184579 | 2^x^e^-28^ | structural molecule activity |
| GO496176 | putative cement protein RIM36 - *Rhipicephalus appendiculatus* (AAK98794.1)  b_microplus|U92790 | 1^x^e^-130^ | structural molecule activity |
| GO496177 | hypothetical protein PGUG_00176 - *Pichia guilliermondii* ATCC 6260 (XP_001486799.1) | 2^x^e^-04^ | structural molecule activity |
| GO496178 | oxidoreductase domain protein - *Enterobacter sp*. 638 (YP_001175795.1)  b_microplus|TC17531 | 3^x^e^+0^ | catalytic activity |
| GO496179 | cytochrome c oxidase subunit III - *Rhipicephalus sanguineus*  (NP_008515.1)  b_microplus|TC17087 | 5^x^e^-38^ | catalytic activity |
| GO496180 | AC002304_31 F14J16.10 - *Arabidopsis thaliana* (AAF79338.1)  b_microplus|TC22130 | 3^x^e^-01^ | catalytic activity |
| GO496181 | enhancer of Polycomb CG7776-PA, isoform A – *Apis mellifera* (XP_397232.1) | 6^x^e^-79^ | transcription regulatory activity |
| GO496182 | hypothetical protein OsI_035683 - *Oryza sativa* (EAY81724.1)  b_microplus|TC22921 | 6^x^e^-04^ | transcription regulatory activity |
| GO496183 | Predicted gene, EG381818 - *Mus musculus* (AAH69935.1)  b_microplus|CV449693 | 1^x^e^-12^ | molecular function unknown |
| GO496184 | putative secreted protein - *Ixodes scapularis* (AAY66614.1)  b_microplus|CV440197 | 3^x^e^-21^ | molecular function unknown |
| GO496185 | hypothetical protein DDBDRAFT_0190925 - *Dictyostelium discoideum* AX4 (XP_646663.1)  b_microplus|TC22779 | 1.3^x^e^-01^ | molecular function unknown |
| GO496186 | hypothetical protein MAL7P1.142 - *Plasmodium falciparum* 3D7  (XP_001349158.1)  b_microplus|TC24862 | 2^x^e^-20^ | molecular function unknown |
| GO496187 | putative secreted protein - *Ixodes scapularis* (AAY66614.1)  b_microplus|CV440198 | 4^x^e^-03^ | molecular function unknown |
| GO496188 | hypothetical protein - *Arabidopsis thaliana* (AAC17072.1) | 3^x^e^-01^ | molecular function unknown |
| GO496189 | microneme-rhoptry antigen - *Theileria annulata* strain Ankara (XP_953099.1) | 5^x^e^-05^ | molecular function unknown |
| GO496190 | putative secreted protein - *Ixodes scapularis* (AAY66614.1) | 7^x^e^-03^ | molecular function unknown |
| GO496191 | hypothetical protein - *Thermobia domestica* (CAM36311.1) | 8^x^e^-06^ | molecular function unknown |
| GO496192 | Putative lipoprotein - *Burkholderia xenovorans* LB400 (YP_560200.1)  b_microplus|TC19098 | 6^x^e^-16^ | molecular function unknown |
| GO496193 | hypothetical protein Kpol_464p3 - *Vanderwaltozyma polyspora* DSM 70294 (XP_001642230.1)  b_microplus|CV440198 | 3^x^e^-01^ | molecular function unknown |
| GO496194 | hypothetical protein - *Thermobia domestica* (CAM36311.1)  b_microplus|TC20527 | 1^x^e^-05^ | molecular function unknown |
| GO496195 | unnamed protein product - *Tetraodon nigroviridis* (CAG11595.1)  b_microplus|TC16479 | 7.5^x^e^-01^ | molecular function unknown |
| GO496196 | hypothetical protein - *Magnetospirillum gryphiswaldense* MSR-1 (CAM73970.1) | 2.3^x^e^-02^ | molecular function unknown |
| GO496197 | putative secreted salivary gland peptide - *Ixodes scapularis* (AAV80791.1)  b_microplus|TC21841 | 2^x^e^-34^ | molecular function unknown |
| GO496198 | AGAP009763-PA - *Anopheles gambiae* str. PEST (EAA14477.4)  b_microplus|TC21159 | 2.7^x^e^-02^ | molecular function unknown |
| GO496199 | MGC79481 protein - *Xenopus tropicalis* (NP_001005003.1) | 1.7^x^e^-01^ | molecular function unknown |
| GO496200 | hypothetical protein - *Equus caballus* (XP_001494787.1)  b_microplus|CK172681 | 9^x^e^-03^ | molecular function unknown |
| GO496201 | salivary mucin with chitin-binding domain - *Argas monolakensis* (ABI52758.1) | 1^x^e^-07^ | molecular function unknown |
| GO496202 | A Chain A, Structure Of Iodinated Cbm25 Amylase - *Bacillus halodurans* (82408236)  b_microplus|TC21438 | 6.2^x^e^-01^ | molecular function unknown |
| GO496203 | hypothetical protein - *Pan troglodytes* (XP_001151054.1)  b_microplus|CK178776 | 1.7^x^e^-01^ | molecular function unknown |
| GO496204 | predicted protein - *Aspergillus terreus* NIH2624 (XP_001213801.1) | 1.8^x^e^-01^ | molecular function unknown |
| GO496205 | conserved hypothetical protein - *Beggiatoa sp*. SS (ZP_01998230.1) | 6^x^e^-12^ | molecular function unknown |
| GO496206 | salivary secreted basic tail protein - *Ornithodoros parkeri* (ABR23390.1)  b_microplus|TC19863 | 2^x^e^-10^ | molecular function unknown |
| **Down-regulated in infected salivary glands** | | | |
| GO496207 | Female-specific histamine-binding protein 1 precursor - *Rhipicephalus appendiculatus* (O77420)  b_microplus|TC18188 | 1^x^e^-23^ | binding |
| GO496208 | immunoglobulin G binding protein C - *Rhipicephalus appendiculatus* (AAB68803.1)  b_microplus|TC20577 | 1^x^e^-81^ | binding |
| GO496209 | serotonin and histamine binding protein - *Dermacentor reticulatus* (AAL56644.1) | 5^x^e^-12^ | binding |
| GO496210 | Kunitz-like protease inhibitor precursor - *Ancylostoma caninum* (AAN10061.1)  b_microplus|TC20102 | 6^x^e^-22^ | binding |
| GO496211 | Hypothetical proteinT20B6.3 - *Caenorhabditis elegans* (NP_497637.1)  b_microplus|TC23476 | 1^x^e^-10^ | binding |
| GO496212 | GR-RBP3 (glycine-rich RNA-binding protein 3) - *Arabidopsis thaliana* (NP_200911.1)  b_microplus|TC20164 | 1^x^e^-07^ | binding |
| GO496213 | proline-rich protein BstNI subfamily 3 precursor - *Homo sapiens* (NP_006240.4) | 2^x^e^-20^ | binding |
| GO496214 | PRB2_HUMAN Basic salivary proline-rich protein - *Homo sapiens* (P02812) | 6^x^e^-24^ | binding |
| GO496215 | plus agglutinin - *Chlamydomonas incerta* (AAX33674.1)  b_microplus|TC15758 | 2^x^e^-15^ | binding |
| GO496216 | minus agglutinin - *Chlamydomonas incerta* (AAW51128.1)  b_microplus|TC19098 | 4^x^e^-05^ | binding |
| GO496217 | keratin associated protein 19-3 - *Homo sapiens* (EAX09897.1)  b_microplus|TC21957 | 7^x^e^-01^ | structural molecule activity |
| GO496218 | CG6004-PB - *Drosophila melanogaster* (NP_648504.2)  b_microplus|CV452616 | 9^x^e^-06^ | structural molecule activity |
| GO496219 | flagelliform silk protein - *Nephila madagascariensis* (AAF36091.1)  b_microplus|TC23771 | 1^x^e^-22^ | structural molecule activity |
| GO496220 | RS29_IXOSC 40S ribosomal protein S29 ribosomal protein S29 - *Ixodes scapularis* (Q4PM47) | 3^x^e^-28^ | structural molecule activity |
| GO496221 | Basic proline-rich protein - *Sus scrofa* (AAK61382.1) | 1^x^e^-21^ | structural molecule activity |
| GO496222 | cement-like antigen – *Haemaphysalis longicornis* (BAF35848.1)  b_microplus|TC1909 | 2^x^e^-07^ | structural molecule activity |
| GO496223 | Putative cement protein RIM36 - *Rhipicephalus appendiculatus* (AAK98794.1)  b_microplus|TC17863 | 1^x^e^-139^ | structural molecule activity |
| GO496224 | Putative cement protein RIM36 - *Rhipicephalus appendiculatus* (AAK98794.1) | 1^x^e^-145^ | structural molecule activity |
| GO496225 | Putative cement protein RIM36 - *Rhipicephalus appendiculatus* (AAK98794.1) | 1^x^e^-151^ | structural molecule activity |
| GO496226 | salivary gland-associated protein 64P - *Rhipicephalus appendiculatus* (AAM09648.1)  b_microplus|TC18640 | 9^x^e^-48^ | structural molecule activity |
| GO496227 | putative cement protein RIM36 - *Rhipicephalus appendiculatus* (AAK98794.1)  b_microplus|TC24800 | 2^x^e^-39^ | structural molecule activity |
| GO496228 | PRB1L precursor protein - *Homo sapiens* (AAB27289.1) | 4^x^e^-13^ | catalytic activity |
| GO496229 | dipeptidyl peptidase - *Nasonia vitripennis* (XP_001601820.1)  b_microplus|TC20123 | 5^x^e^-07^ | catalytic activity |
| GO496230 | matrix metalloproteinase 1 (interstitial collagenase) - *Xenopus laevis* (NP_001080518.1) | 7^x^e^-01^ | catalytic activity |
| GO496231 | Salivary gland metalloprotease - *Boophilus microplus* (AAZ39660.1)  b_microplus|TC18456 | 1^x^e^-50^ | catalytic activity |
| GO496232 | 26kDa protease - *Sarcophaga peregrine* (BAA22400.1)  b_microplus|TC17022 | 1^x^e^-17^ | catalytic activity |
| GO496233 | invertase/pectin methylesterase inhibitor family protein - *Arabidopsis thaliana* (NP_176463.2)  b_microplus|TC16199 | 1^x^e^-01^ | enzyme regulatory activity |
| GO496234 | homeobox B1 - *Mus musculus* (NP_032292.2)  b_microplus|TC17118 | 1^x^e^-01^ | transporter activity |
| GO496235 | mannose dehydratase, NAD(P)-binding - *Frankia aln* (YP_712286.1)  b_microplus|TC22512 | 3^x^e^-04^ | transporter activity |
| GO496236 | surface-erythrocyte phosphoprotein - *Babesia rossi* (CAD10043.1)  b_microplus|TC23771 | 5^x^e^-01^ | transporter activity |
| GO496237 | conserved hypothetical protein - *Trichomonas vaginalis* G3 (XP_001318272.1)  b_microplus|TC21871 | 4^x^e^-08^ | molecular function unknown |
| GO496238 | hypothetical protein FRAAL1158 - *Frankia alni* ACN14a (YP_712532.1)  b_microplus|TC21414 | 1^x^e^-04^ | molecular function unknown |
| GO496239 | hypothetical protein EhV364 - *Emiliania huxleyi* virus 86 (YP_294122.1) | 9^x^e^-23^ | molecular function unknown |
| GO496240 | cell surface SD repeat antigen precursor, putative - *Streptococcus sanguinis* SK36 (YP_001035905.1)  b_microplus|TC16475 | 3^x^e^-06^ | molecular function unknown |
| GO496241 | hypothetical protein -*Strongylocentrotus purpuratus* (XP_783923.2)  b_microplus|CK176028 | 5^x^e^-06^ | molecular function unknown |
| GO496242 | SJCHGC03140 protein - *Schistosoma japonicum* (AAX26495.2) | 4.7^x^e^-01^ | molecular function unknown |
| GO496243 | hypothetical protein; putative membrane protein - *Frankia alni* ACN14a (YP_712532.1)  b_microplus|TC17022 | 1.1^x^e^+00^ | molecular function unknown |
| GO496244 | SET domain containing 1B -*Homo sapiens* (XP_946855.2)  b_microplus|TC20104 | 1.5^x^e^-01^ | molecular function unknown |
| GO496245 | hypothetical protein GLP_609_40294_39134 -*Giardia lamblia* (XP_770752.1)  b_microplus|TC19098 | 9.3^x^e^-01^ | molecular function unknown |
| GO496246 | hCG1793893 - *Homo sapiens* (EAX07174.1) | 5^x^e^-24^ | molecular function unknown |
| GO496247 | hypothetical protein -*Cryptococcus neoformans var. neoformans* JEC21 (XP_569355.1)  b_microplus|TC21386 | 3.3^x^e^+00^ | molecular function unknown |
| GO496248 | hypothetical protein TTHERM_00849310 -*Tetrahymena thermophila* SB210 (XP_001019233.1) | 5.5^x^e^-01^ | molecular function unknown |
| GO496249 | unnamed protein product -*Candida glabrata* (XP_448999.1)  b_microplus|TC21702 | 4^x^e^-03^ | molecular function unknown |
| GO496250 | hypothetical protein OsJ_010161 - *Oryza sativa* (EAZ26678.1)  b_microplus|TC16663 | 3^x^e^-12^ | molecular function unknown |
| GO496251 | hypothetical protein - *Rattus norvegicus* (XP_001054782.1)  b_microplus|TC17460 | 2^x^e^-09^ | molecular function unknown |
| GO496252 | hypothetical protein - *Monodelphis domestica* (XP_001376188.1) | 2^x^e^-05^ | molecular function unknown |
| GO496253 | PRB3 protein - *Homo sapiens* (AAH96211.1)  b_microplus|TC20839 | 4^x^e^-06^ | molecular function unknown |
| GO496254 | predicted protein - *Nematostella vectensis* (XP_001638632.1) | 2^x^e^-01^ | molecular function unknown |
| GO496255 | unnamed protein product - *Tetraodon nigroviridis* (CAF94182.1) | 1.5^x^e^+00^ | molecular function unknown |
| GO496256 | predicted protein - *Sclerotinia sclerotiorum*  (XP_001596482.1) | 2.5^x^e^+00^ | molecular function unknown |
| GO496257 | Period gamma - *Apis cerana* (BAD06463.1) | 1.4^x^e^+00^ | molecular function unknown |
| GO496258 | hypothetical protein - *Haemaphysalis longicornis* (BAE02553.1)  b_microplus|TC21071 | 1^x^e^-10^ | molecular function unknown |
| GO496259 | hypothetical protein 3 - *Microplitis demolitor bracovirus* (YP_239367.1)  b_microplus|TC16665 | 1^x^e^-03^ | molecular function unknown |
| GO496260 | 20/24 kDa immunodominant saliva protein - *Rhipicephalus appendiculatus* (AAO60049.1)  b_microplus|CV444142 | 7^x^e^-08^ | molecular function unknown |
| GO496261 | unnamed protein product - *Homo sapiens* (BAC86958.1)  b_microplus|TC15758 | 1.4^x^e^-02^ | molecular function unknown |
| GO496262 | hypothetical protein PC104124.00.0-*Plasmodium chabaudi chabaudi* (XP_743423.1) | 3^x^e^-01^ | molecular function unknown |
