# Supplementary material for: Differential expression of genes in salivary glands of male Rhipicephalus (Boophilus)microplus in response to infection with Anaplasma marginale
Source: BMC Genomics. 2010 Mar 18;11:186. doi: 10.1186/1471-2164-11-186 (PMC2848250; doi:10.1186/1471-2164-11-186)
Supplement: Additional file 2 — Differentially expressed genes selected based on their putative role during A. marginale infection to validate SSH results by real-time RT-PCR. [file 1471-2164-11-186-S2.DOC]

**Table S2-** Differentially expressed genes selected based on their putative role during *A. marginale* infection to validate SSH results by real-time RT-PCR

| **EST** | **Sequence identity** | **Up- or Down- regulated in infected salivary glands** | **Short description** |
| --- | --- | --- | --- |
| 7BstNI3 | *Homo sapiens* (NP_006240.4) | down | proline-rich protein BstNI subfamily 3 precursor |
| 9PRB2 | *Homo sapiens* (P02812) | down | PRB2_HUMAN Basic salivary proline-rich protein |
| 22Hbp | *Rhipicephalus appendiculatus* (077420) | down | female-specific histamine-binding protein 1 precursor |
| 28ImbpC | *R. appendiculatus* (AAB68803.1) | down | immunoglobulin G binding protein C |
| 59Rib | *Ixodes scapularis* (Q4PM47) | down | ribosomal protein S29 mRNA |
| 100Silk | *Nephila madagascariensis* (AAF36091.1) | down | flagelliform silk protein |
| 104SrHb | *Dermacentor reticulatus* (AAL566441) | down | serotonin and histamine binding protein |
| 108Kunz | *Ancylostoma caninum* (AAN10061.1) | down | Kunitz-like protease inhibitor precursor |
| 120Ptse | *Sarcophaga peregrine* (BAA22400.1) | down | 26kDa protease |
| 128PecIn | *Arabidopsis thaliana* (NP_176463.2) | down | invertase/pectin methylesterase inhibitor family protein |
| 36vATP | *Aedes aegypti* (XP_001600097.1) | up | vacuolar H+-ATPase V1 sector subunit |
| 88BstNI | *Homo sapiens* (NP_005030.2) | up | proline-rich protein BstNI subfamily 1 isoform 1 |
| 93Meth | *Oscillatoria brevis* (BAC76027.1) | up | metallothionein |
| 94Will | *Ixodes ricinus* (AAQ01562.1) | up | von Willebrand factor mRNA |
